# Supplementary material for: A stochastic modelling framework for cancer patient trajectories: combining tumour growth, metastasis, and survival
Source: J Math Biol. 2025 May 22;90(6):65. doi: 10.1007/s00285-025-02229-6 (PMC12098478; doi:10.1007/s00285-025-02229-6)
Supplement: Supplementary file 1 — (pdf 1664 KB) [file 285_2025_2229_MOESM1_ESM.pdf]

Supplementary Information: A Stochastic Modelling Framework for Cancer Patient Trajectories: Combining Tumour Growth, Metastasis, and Survival

Vincent Wieland and Jan Hasenauer

A Measurement Noise and Survival Time Distributions

A.1 Log-Normal Measurement Noise

In the simulation study shown in the main manuscript, we only considered Gaussian noise for the tumour size measurements. In general, the framework exhibits enough flexibility to deal with various noise formulations and the precise noise model that is used has to depend on the dataset at hand. To showcase this ability of our framework, we provide here the results for a experiment using the exponential proportional model together with log-normal distributed tumour size measurement noise, i.e.

$$\tilde{S}(t) \sim \text{LogNormal}(\log(S(t)), \sigma), \qquad \text{where} \quad \sigma = 1.0.$$

The optimization still showed good convergence, Supplementary Figure S14 and parameter estimates and corresponding credibility intervals covered the true values, highlighting the accuracy of the retrieved estimates, Supplementary Figure S15.

A.2 Survival Time Distributions

Here we provide the parametrizations of the three survival time distribution as used in the manuscript.

Exponential Distribution

The exponential distribution is the simplest and continuous analogue of the geometric distribution. It's key property is the memoryless property which relates to having a constant hazard. We use the parametrization depending on a scale parameter $\lambda > 0$ and probability density function

$$f(x|\lambda) = \begin{cases} \lambda e^{-\lambda x}, & x \geq 0 \\ 0, & x < 0 \end{cases}$$

Weibull Distribution

The Weibull distribution extends the exponential distribution by relaxing the assumption of constant hazard. Using a shape parameter $k > 0$ and a scale parameter $\lambda > 0$ the probability density function is given by

$$f(x|\lambda, k) = \begin{cases} \frac{k}{\lambda} \left(\frac{x}{\lambda}\right)^{k-1} e^{-(x/\lambda)^k}, & x \geq 0 \\ 0, & x < 0 \end{cases}$$

A value of $k < 1$ corresponds to a decreasing hazard over time and $k > 1$ to an increasing hazard over time, e.g. due to aging. In the case of $k = 1$ the Weibull distribution reduces to the exponential distribution with scale λ.

Generalized Gamma Distribution

We use the parametrization with two shape parameters $d > 0, p > 0$ and a scale parameter $a > 0$ and support $x \in (0, \infty)$. The probability density function is then given by

$$f(x|a, d, p) = \frac{(p/a^d)x^{d-1}e^{-(x/a)^p}}{\Gamma(d/p)},$$

where $\Gamma(\cdot)$ denotes the gamma function.
This distribution generalizes the gamma distribution which has one shape parameter and also includes as special cases the exponential distribution, i.e. $d = p = 1$ and $\lambda = a$, and the Weibull distribution, i.e. $k = d = p$ and $\lambda = a$.

B Supplementary Figures

(a) (b)

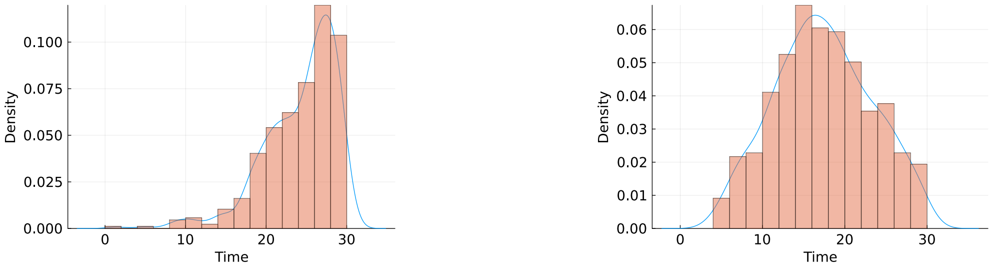

Fig. S1: **Overall survival time distributions.** Histograms and kernel density estimates (blue lines) of the empirical distribution of overall survival times observed in the synthetic data sets for the exponential proportional model (M2) in S1a and the Gompertz model (M3) in S1b.

(a) Exponential growth based model. (b) Gompertz growth based model.

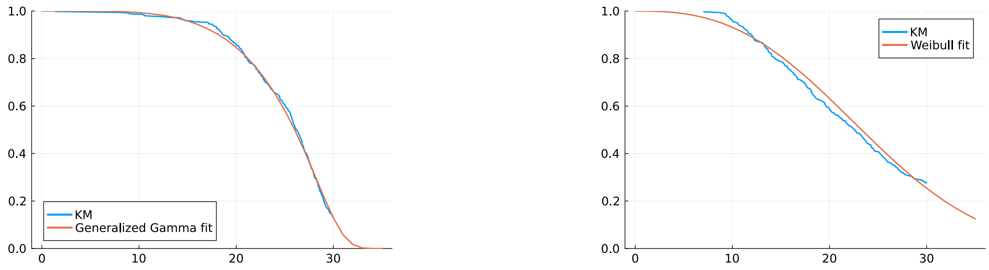

Fig. S2: **Assessment of survival curves.**Kaplan-Meier estimator (KM) for the survival curve of the overall survival observed in the synthetic data (blue) plotted against the survival curve of a fitted distribution (orange). For S2a we used a generalized Gamma distribution with $a = 4.5, p = 19.5, d = 30.9$ and the exponential proportional model (M1), for S2b a Weibull distribution with $k = 2.6, \lambda = 21.9$ and the Gompertz model (M3).

(a) SAMIN - Numeric (b) SAMIN - Analytic (c) LBFGS - Analytic

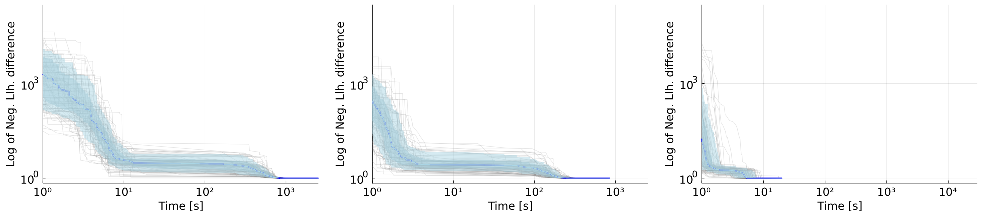

Fig. S3: **Optimization of the cell-division model.** Optimiser traces of the current best negative log likelihood value over time for 100 single optimisation runs of the cell division model.

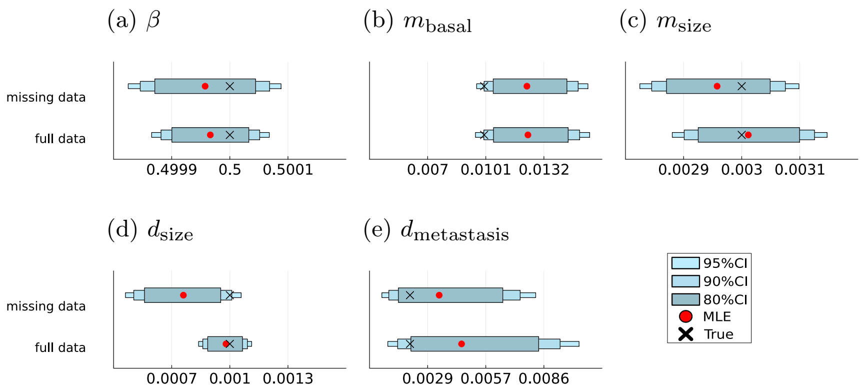

Fig. S4: **Parameter inference results for the exponential proportional model.** Maximum likelihood estimates (MLE) and sampling-based credibility intervals for the model parameters of the exponential proportional model with the full data set and with an unregular data set, where we randomly subsampled 70% of the datapoints and added 20% of missingness into the tumour and the metastasis measurements each.

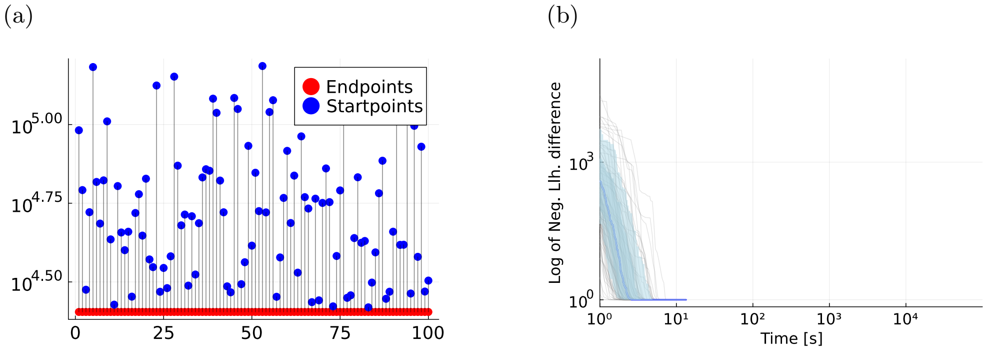

Fig. S5: **Optimization of the exponential proportional model with unregular data.** Evaluation of 100 starts of LBFGS optimiser using analytical likelihoods and the exponential proportional model with an unregular data set, where we randomly subsampled 70% of the datapoints and added 20% of missingness into the tumour and the metastasis measurements each. S5a shows the double waterfall plot and S5b visualizes the optimizer traces.

(a) Exponential proportional model

(b) Cell division model

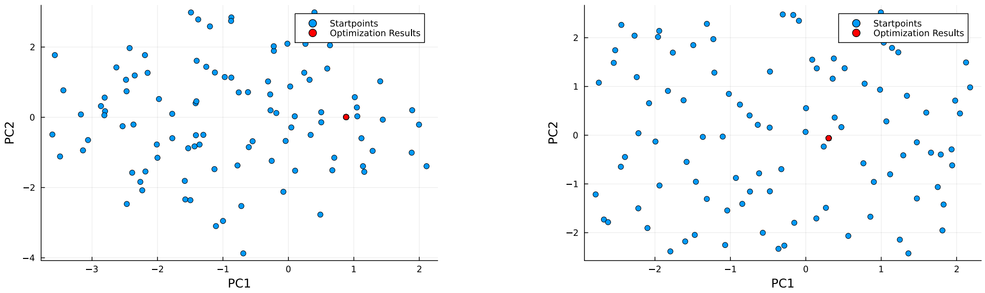

Fig. S6: **Illustration of optimization start- and endpoints.** Visualization of the randomly sampled startpoints and the optimisation endpoint by a PCA on the parameter space.

(a) Exponential proportional model - Analytic likelihood

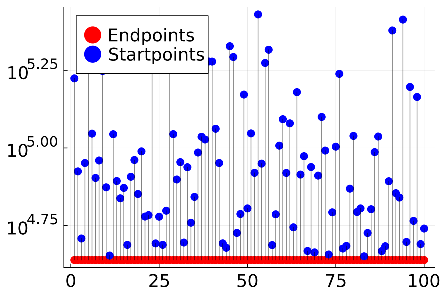

(b) Cell division model - Analytic likelihood

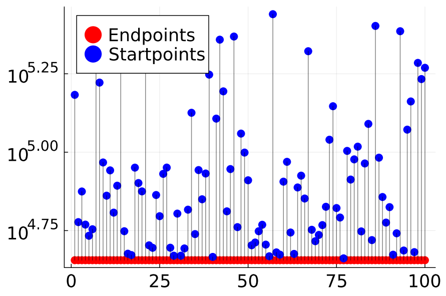

(c) Exponential proportional model - Numeric likelihood

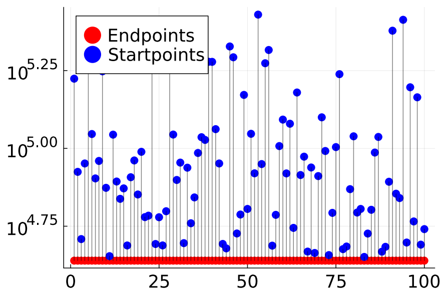

(d) Cell division model - Numeric likelihoods

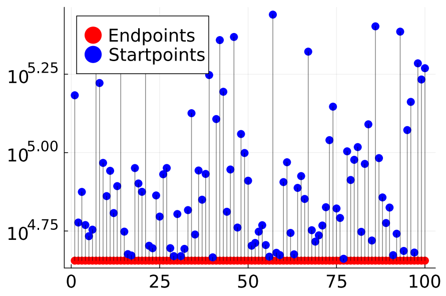

Fig. S7: **Convergence of SAMIN optimizations.** Waterfall plots indicating the negative log-likelihood value of the startpoints and the corresponding endpoint of the optimisation. The plots where retrieved from the estimation results with analytical likelihoods and the SAMIN optimiser.

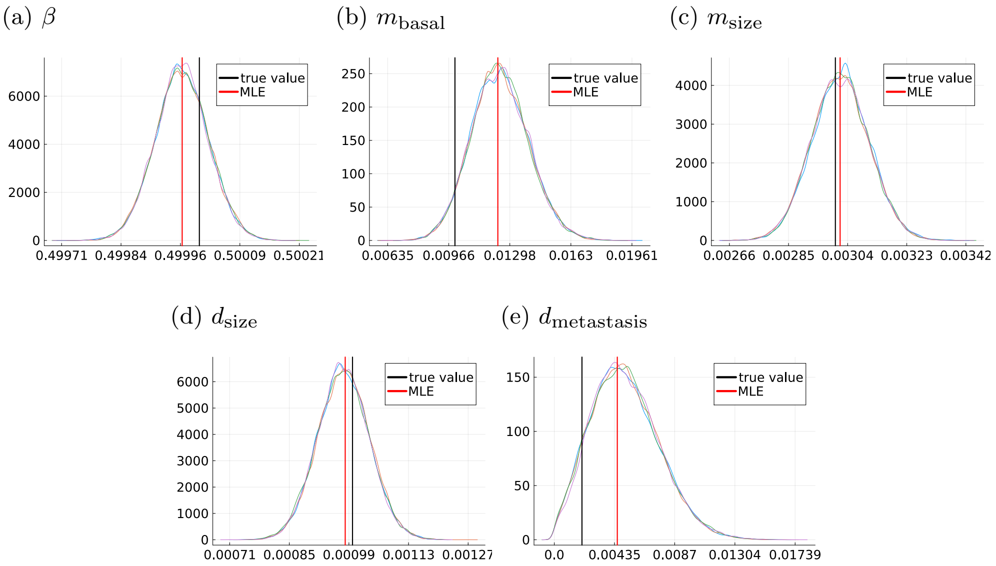

Fig. S8: **Sampling results of the exponential proportional model.** MCMC sampling results visualized by density estimates over the samples for the model parameters of the exponential proportional model. The maximum likelihood estimator and the parameter value underlying the simulated data are indicated by vertical lines.

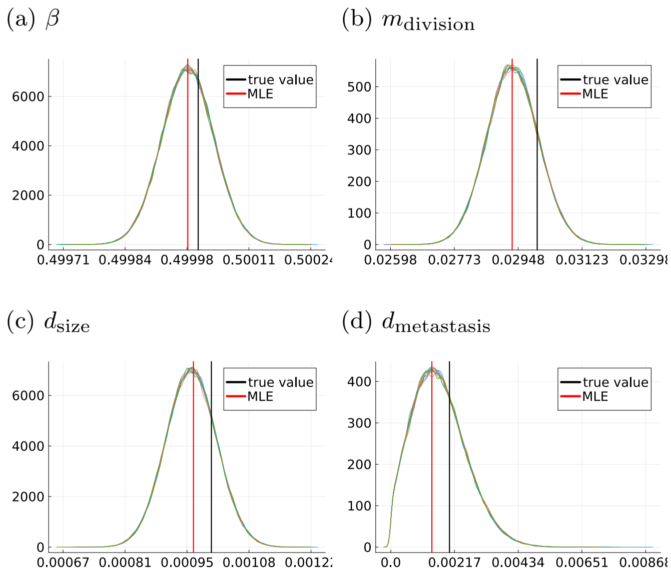

Fig. S9: **Sampling results of the cell-division model.** MCMC sampling results visualized by density estimates for the model parameters of the cell-division model. The maximum likelihood estimator and the parameter value underlying the simulated data are indicated by vertical lines.

(a) Proportional data and proportional model

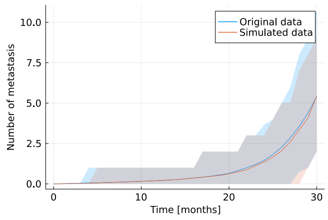

(b) Proportional data and cell division model

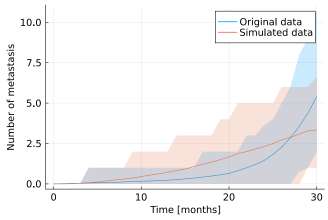

(c) Cell division data and proportional model.

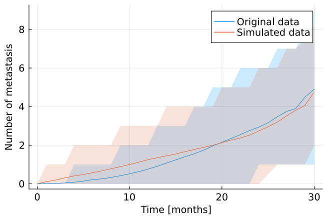

(d) Cell division data and cell division model.

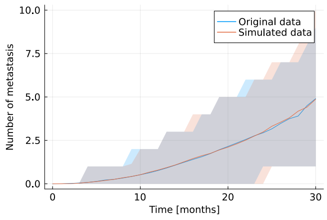

Fig. S10: **Model selection results.** Model fits for the selection of the metastasis processes. We plotted the mean trajectory and a 95% confidence interval from the dataset used for the optimisation (blue) and for the data simulated with the model and corresponding MLE of the model parameters (orange). The boxes indicate the models with the smallest AIC value.

(a)

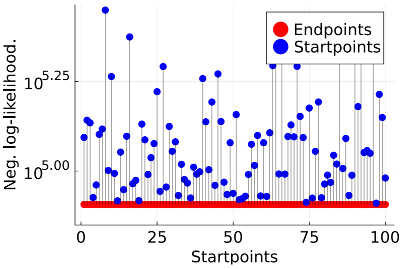

(b)

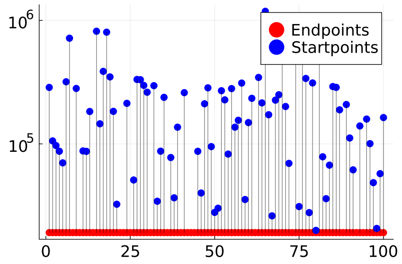

Fig. S11: Double waterfall plot for the evaluation of the optimisation runs for S11a Gompertz model and S11b the Gyllenberg-Webb model. The plot shows the start- and endpoints of each optimisation run.

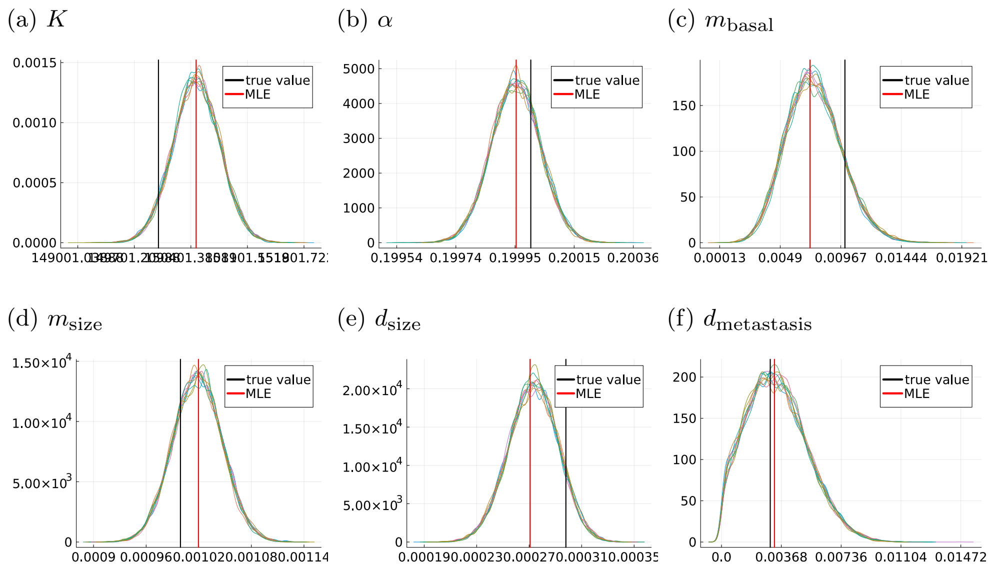

Fig. S12: **Sampling results of the Gompertz model.** Estimation and sampling results visualized by density estimates for the model parameters of the Gompertz model. The maximum likelihood estimator and the parameter value underlying the simulated data are indicated by vertical lines.

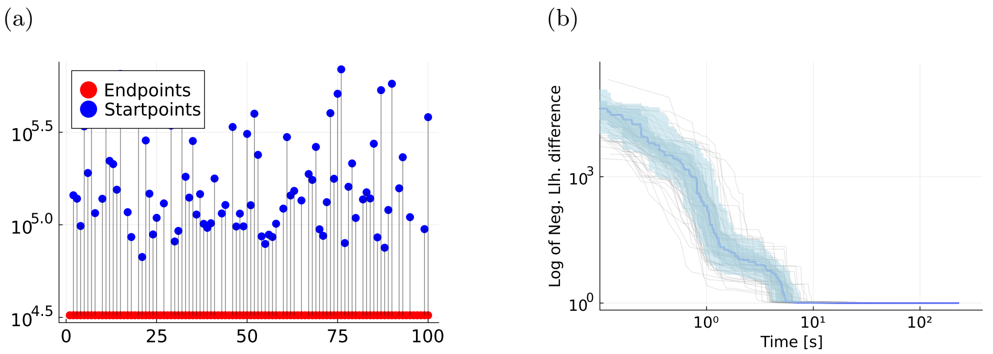

Fig. S13: **Optimization of the treatment effect model.** Evaluation of 100 optimization runs of the treatment effect model. S13a shows the double waterfall plot and S13b visualizes the optimizer traces.

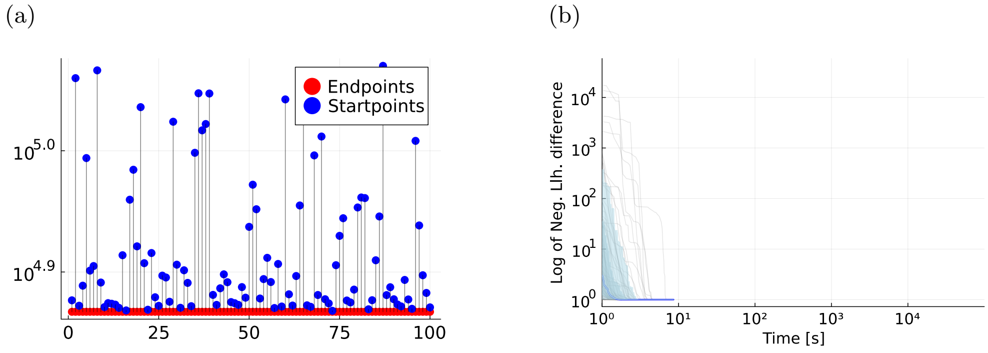

Fig. S14: **Optimization of the lognormal noise model.** Evaluation of 100 starts of LBFGS optimiser using analytical likelihoods and the exponential proportional model with a lognormal noise model for the tumour size measurements. S14a shows the double waterfall plot and S14b visualizes the optimizer traces.

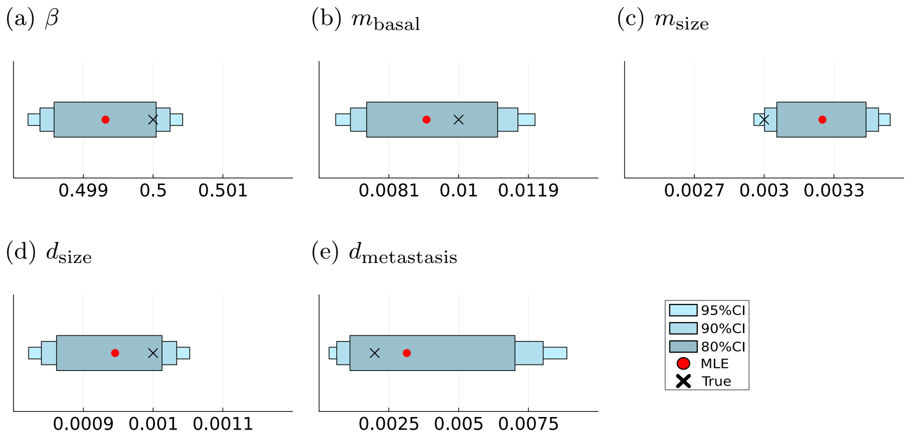

Fig. S15: **Parameter inference results for the lognormal noise model.** Maximum likelihood estimates (MLE) and sampling-based credibility intervals for the model parameters of the exponential proportional model with log-normal measurement noise for the tumour size measurements.

C Supplementary Tables

Table S1: Model parameters used in the model (M2) with exponential growth and cell division based metastasis spread in Section 2.4 to mimick the data characteristics from Engel et al (2003).

Parameter	Value
β	0.01
m_{division}	6.0
d_{size}	0.011
$d_{\text{metastasis}}$	0.31

Table S2: Model parameters, their true values and respective ranges for exponential growth based models on log-scale.

Parameter	True value	Parameter range
β	-0.693	$[-0.75, -0.65]$
m_{basal}	-4.605	$[-7, -2]$
m_{size}	-5.809	$[-7, -2]$
m_{division}	-3.506	$[-7, -2]$
d_{size}	-6.908	$[-9, -4]$
$d_{\text{metastasis}}$	-6.215	$[-9, -4]$

Table S3: Mean evaluation times for the function evaluation of the negative log-likelihood function. The mean was taken over the evaluation times of 100 randomly sampled parameter vectors.

Model / Likelihood type	Analytical likelihood	Numerical likelihood
Exponential proportional	14.9 ms	893.9 ms
Cell division	24.3 ms	102.6 ms

Table S4: Mean run-time of the optimisation. The mean was taken over 100 optimisation runs initialized at 100 randomly sampled startpoints.

Model / Llh type and optimiser	Analytical - LBFGS	Analytical - SAMIN	Numerical - SAMIN
Exponential proportional	13.3 s	501.1 s	26565.6 s
Cell division	5.8	684.4 s	2375.1 s

Table S5: Exponential proportional model estimation results.

Parameter	True value	Estimate (95%-CI)
β	0.5	0.4999 (0.4998; 0.5)
m_{basal}	0.01	0.009147 (0.0067; 0.0125)
m_{size}	0.003	0.0029 (0.0027; 0.0031)
d_{size}	0.001	0.00097 (0.0008; 0.0011)
$d_{\text{metastasis}}$	0.002	0.0035 (0.0003; 0.0103)

Table S6: Cell division model estimation results.

Parameter	True value	Estimate (95%-CI)
β	0.5	0.49998 (0.4999; 0.5001)
m_{division}	0.03	0.0293 (0.028; 0.0307)
d_{size}	0.001	0.00096 (0.0008; 0.0011)
$d_{\text{metastasis}}$	0.002	0.0014 (0.0002; 0.0037)

Table S7: Log-scale parameter values and bounds for the Gompertz (S7a) and Gyllenberg–Webb (S7b) models.

(a)

Parameter	True value	Parameter range
K	11.918	$[11.849, 11.983]$
α	-1.609	$[-1.5, -1.75]$
m_{basal}	-4.605	$[-7, -2]$
m_{size}	-6.908	$[-7, -2]$
d_{size}	-8.1117	$[-9, -4]$
$d_{\text{metastasis}}$	-5.809	$[-9, -4]$

(b)

Parameter	True value	Parameter range
b	0.0	$[-1.0, -1.0]$
μ	-2.996	$[-4.0, 0.0]$
m_{basal}	-3.219	$[-7, -2]$
m_{size}	-3.219	$[-7, -2]$
d_{size}	-4.605	$[-9, -4]$
$d_{\text{metastasis}}$	-4.605	$[-9, -4]$

D Proofs

In this supporting section we give the proofs for Theorem 1 and Corollary 2.

Proof of Theorem 1

Proof. For notational simplicity we do not explicitly write the dependence on other processes for the intensity rates and write $\lambda_N(t), \Lambda_N([t_{j-1}, t_j)), \lambda_D(t), \Lambda_D([t_{j-1}, t_j))$. Additionally, we denote with $\Lambda_D([t_{j-1}, t_j), n)$ the accumulated death process intensity function for a time-interval, where the number of metastasis n is constant.

Let us first consider the simpler case of $m = 0$ new metastasis in the time interval $[t_{j-1}, t_j)$ and $D(t_j) = D(t_{j-1}) = 0$, the patient survived during the time interval of interest. Then by the property of exponentially distributed waiting times in a Poisson process, the likelihood contribution is given by.

$$\mathbb{P}(D(t_j) = 0 \mid D(t_{j-1}) = 0,\ N(t_{j-1}) = \hat{n},\ N(t_j) = \hat{n})$$

$$
\begin{aligned}
&=\mathbb{P}\left(\int_{t_{j-1}}^{t_j} d_{basal} + d_{\text{size}}\sqrt{S(s)} + d_{\text{metas}}\hat{n} ds < Y \sim \text{Exp}(1)\right)\\
&=\exp\left(-\int_{t_{j-1}}^{t_j} d_{basal} + d_{\text{size}}\sqrt{S(s)} + d_{\text{metas}}\hat{n} ds\right)\\
&=e^{-\Lambda_D([t_{j-1},t_j),\hat{n})}
\end{aligned}
$$

For shorter notation we name the solution of the last integral by

$$
\begin{aligned}
\Psi([t_{j-1},t_j),n) :&= e^{-\Lambda_D([t_{j-1},t_j),n)}\\
&= \exp\left(-\int_{t_{j-1}}^{t_j} d_{basal} + d_{\text{size}}\sqrt{S(s)} + d_{\text{metas}} n ds\right)\\
&= e^{-(t_j-t_{j-1})\cdot(d_{\text{basal}}+\hat{n}d_{\text{metas}})}\cdot\exp\left(-\frac{2d_{\text{size}}}{\beta}(\sqrt{S(t_j)}-\sqrt{S(t_{j-1})})\right)
\end{aligned}
\tag{1}
$$

If we observe $m = 1$ new metastasis in the time interval of interest, we can simply split the interval at that timepoint u, where the metastasis occurred and get

$$
\begin{aligned}
&\mathbb{P}(D(t_j) = 0|D(t_{j-1}) = 0, N(t_{j-1}) = \hat{n}, N(t_j) = \hat{n}+1, \text{met at } du)\\
=&\exp\left(-\Lambda_D(t_{j-1},u,\hat{n}) - \Lambda_D(u,t_j,\hat{n}+1)\right)
\end{aligned}
$$

However, since we do not observe this timepoint, we need to integrate over the full time interval with respect to the probability that the metastasis occurred at that timepoint. This probability is given by

$$
\mathbb{P}(\text{met jump at } du) = \lambda_N(u)e^{-\Lambda_N([t_{j-1},t_j))}, \tag{2}
$$

where $\lambda_N(u)$ denotes the instantaneous rate of a jump at u

$$
\lambda_N(u) = \lim_{\varepsilon\to 0}\frac{\mathbb{P}(X_{u+\varepsilon} > X_u)}{\varepsilon}
$$

and $e^{-\Lambda_N([t_{j-1},t_j))}$ is the normalization constant based on the mean of jumps in the interval[1]

$$
e^{-\mathbb{E}[N(t_{j-1},t_j)]} = e^{-\Lambda_N([t_{j-1},t_j))} = e^{-\int_0^{\Delta t}\lambda_N(t_{j-1}+s)ds}.
$$

However, this omits the information that we already conditioned on having exactly one jump in the interval, so we need to divide by that probability.

[1]Compare to eq. 16.13 in (Gabbiani and Cox 2010)

$$\mathbb{P}(\text{met at } du|1 \text{ jump}) = \frac{\lambda_N(u)e^{-\Lambda_N([t_{j-1},t_j])}}{\Lambda_N([t_{j-1},t_j))^1/1! \quad e^{-\Lambda_N([t_{j-1},t_j])}} = \frac{\lambda_N(u)}{\Lambda_N([t_{j-1},t_j))}$$

Together this yields:

$$\begin{aligned}
&\mathbb{P}(D(t_j)=0|D(t_{j-1})=0, N(t_{j-1})=\hat{n}, N(t_j)=\hat{n}+1)\\
&=\int_{t_{j-1}}^{t_j} \mathbb{P}(D(t_j)=0|D(t_{j-1})=0, N(t_{j-1})=\hat{n}, N(t_j)=\hat{n}+1, \text{met at } du)\mathbb{P}(\text{met at } du)\, du\\
&=\int_{t_{j-1}}^{t_j} \exp\left(-\Lambda_D([t_{j-1},t_j))\right)\mathbb{P}(\text{met at } du)du\\
&=\int_{t_{j-1}}^{t_j} \exp\left(-\Lambda_D([t_{j-1},u],\hat{n}) - \Lambda_D([u,t_j],\hat{n}+1)\right)\mathbb{P}(\text{met at } du)du\\
&=\int_{t_{j-1}}^{t_j} \frac{\lambda_N(u)}{\Lambda_N([t_{j-1},t_j))}\Psi([t_{j-1},u],\hat{n})\Psi([u,t_j],\hat{n}+1)du
\end{aligned}$$

Analogously, we get for the case of m new metastasis, $N(t_j) = N(t_{j-1}) + m \equiv \hat{n} + m$.

$$\begin{aligned}
\mathbb{P}(\text{met at } du_1,...,du_m|m \text{ jumps}) &= \frac{\lambda_N(u_1)...\lambda_N(u_m)e^{-\Lambda_N([t_{j-1},t_j])}}{\Lambda_N([t_{j-1},t_j))^m/m! \quad e^{-\Lambda_N([t_{j-1},t_j])}}\\
&= \frac{m!\lambda_N(u_1)...\lambda_N(u_M)}{\Lambda_N([t_{j-1},t_j))^m}.
\end{aligned}$$

This yields the desired result

$$\begin{aligned}
&\mathbb{P}(D(t_j)=0|D(t_{j-1})=0, N(t_{j-1}), N(t_j))\\
&=\int_{t_{j-1}}^{t_j}\int_{u_1}^{t_j}...\int_{u_m}^{t_j} \frac{m!\,\lambda_N(u_1)...\lambda_N(u_m)}{\Lambda([t_{j-1},t_j))^m}\prod_{i=0}^{m}\Psi(u_i,u_{i+1},\hat{n}+i)du_m...du_1.
\end{aligned} \tag{3}$$

□

Proof of Corollary 2

Proof. For the case $D(t_j) = 1, D(t_{j-1}) = 0$, we now that death occurs exactly at time t_j. So, we need to get the density for the time until an event given we did not see it before.

By the definition of a non-homogeneous Poisson process we know

$$\mathbb{P}(D(t_j) - D(t_{j-1}) = n) = \frac{\Lambda_D([t_{j-1},t_j))^n}{n!}\exp(-\Lambda_D([t_{j-1},t_j)))$$

Therefore, we get for the distribution function for the time T until the next event after t_{j-1}

$$
\begin{aligned}
F_T(T \le t_j - t_{j-1}) = 1 - \mathbb{P}(T > t_j - t_{j-1}) &= 1 - \mathbb{P}(D(t_j) - D(t_{j-1}) = 0) \\
&= 1 - \exp(-\Lambda_D([t_{j-1}, t_j)))
\end{aligned}
$$

Differentiating this yields as a density for the time to next event after t_{j-1}

$$
\begin{aligned}
f_T(t) &= \Lambda_D'([t_{j-1}, t_{j-1} + t]) \exp(-\Lambda_D([t_{j-1}, t_{j-1} + t])) \\
&= \lambda_D(t_{j-1} + t) \exp(-\Lambda_D([t_{j-1}, t_{j-1} + t]))
\end{aligned}
$$

Intuitively this is the probability to survive until $t_{j-1} + t$ multiplied with the instantaneous rate of dying at that time.

We then apply Theorem 1 for the form of the survival probability, given that m new metastasis occurred, $N(t_j) = N(t_{j-1}) + m \equiv \hat{n} + m$. This then yields

$$
\begin{aligned}
&\mathbb{P}(D(t_j) = 1 | D(t_{j-1}) = 0, N(t_{j-1}), N(t_j)) \\
&= \int_{t_{j-1}}^{t_j} \int_{u_1}^{t_j} ... \int_{u_m}^{t_j} \frac{m! \, \lambda_N(u_1) ... \lambda_N(u_m)}{\Lambda([t_{j-1}, t_j))^m} \lambda_D(t_j) \prod_{i=0}^{m} \Psi(u_i, u_{i+1}, \hat{n} + i) du_m ... du_1
\end{aligned}
$$

□

E Analytical Likelihood Formulas

In this supplementary part, we provide the precise expressions of the analytically computed likelihood contributions (14) used in the simulation studies in Section 4. We incorporate the assumption of $t_0 = 0$ and make the dependence on parameters explicit in the arguments of the functions.

Cell Division Model

Given the following functions for tumour growth and intensity rates of the Poisson point processes

$$
\begin{aligned}
S(t, \beta) &= S_0 \cdot e^{\beta \cdot t} \\
\lambda_N(t, \beta, m_{\text{division}}) &= m_{\text{division}} \beta \left(\frac{\ln \left(\frac{S_0 \exp(\beta t)}{S_{\text{cell}}} \right)}{\ln(2)} \right)^k = m_{\text{division}} \beta \left(\frac{\beta t}{\ln(2)} \right)^k . \\
\Lambda_N([t_{j-1}, t_j), \beta, m_{\text{division}}) &= \frac{m_{\text{division}}}{((k+1) \ln(2)^k)} \beta^{(k+1)} (t_j^{(k+1)} - t_{j-1}^{(k+1)}) \\
\lambda_D(t, \beta, d_{\text{size}}, d_{\text{metas}}, n) &= d_{\text{size}} \sqrt{S} + d_{\text{metas}} n
\end{aligned}
$$

$$\Lambda_D(t_{j-1}, t_j, \beta, d_{\text{size}}, d_{\text{metas}}) = (t_j - t_{j-1})(n d_{\text{metas}}) + \frac{2 d_{\text{size}} \sqrt{S_0}}{\beta} (\sqrt{e^{\beta t_j}} - \sqrt{e^{\beta t_{j-1}}})$$

the survival probability over an interval $[t_{j-1}, t_j)$ with $N(t_{j-1}) = n$ takes the following functional forms for a given number of new metastasis $m \in \{0, ..., 5\}$.

$$\mathbb{P}(D(t_j) = 0 | D(t_{j-1}) = 0, N(t_{j-1}) = n, N(t_j) = n + m) =$$

$$\frac{\left(\frac{\beta}{d_{\text{metas}}}\right)^m \exp\left(\frac{2 d_{\text{size}} \left(\exp\left(\frac{\beta t_{j-1}}{2}\right) - \exp\left(\frac{\beta t_j}{2}\right)\right) \sqrt{S_0}}{\beta} + d_{\text{metas}} n t_{j-1} - d_{\text{metas}} (n+m) t_j\right)}{(-1)^m \beta^m d_{\text{metas}}^m (t_{j-1}^{k+1} - t_j^{k+1})^m}$$

$$\cdot (k+1)^m (\Gamma(2.0, -(d_{\text{metas}} t_{j-1})) - \Gamma(2.0, -(d_{\text{metas}} t_j)))^m \log(2)^m,$$

where $\Gamma(s, x)$ denotes the upper incomplete gamma function defined as

$$\Gamma(s, x) = \int_x^\infty t^{s-1} e^{-t} dt$$

Exponential Proportional Model

Given the following functions for tumour growth and intensity rates of the Poisson point processes

$$S(t) = S_0 \cdot e^{\beta \cdot t}$$

$$\lambda_N(t, \beta, m_{\text{basal}}, m_{\text{size}}) = m_{\text{basal}} + m_{\text{size}} \sqrt{S_0 \exp(\beta t)}$$

$$\Lambda_N([t_{j-1}, t_j), \beta, m_{\text{basal}}, m_{\text{size}}) = (t_j - t_{j-1}) m_{\text{basal}} + \frac{2 m_{\text{size}} \sqrt{S_0}}{\beta} (\sqrt{e^{\beta t_j}} - \sqrt{e^{\beta t_{j-1}}})$$

$$\lambda_D(t, \beta, d_{\text{size}}, d_{\text{metas}}, n) = d_{\text{size}} \sqrt{S} + d_{\text{metas}} n$$

$$\Lambda_D(t_{j-1}, t_j, \beta, d_{\text{size}}, d_{\text{metas}}) = (t_j - t_{j-1})(n d_{\text{metas}}) + \frac{2 d_{\text{size}} \sqrt{S_0}}{\beta} (\sqrt{e^{\beta t_j}} - \sqrt{e^{\beta t_{j-1}}})$$

the survival probability over an interval $[t_{j-1}, t_j)$ with $N(t_{j-1} = \hat{n}$ takes the following functional forms for different given number of new metastasis $m \in \{0, ..., 5\}$

m=0:

$$\Psi(t_{j-1}, t_j, \beta, d_{\text{size}}, d_{\text{metas}}, S_0, n) = e^{\frac{2 \cdot d_{\text{size}} \cdot (\sqrt{e^{\beta \cdot t_{j-1}} \cdot S_0} - \sqrt{e^{\beta \cdot t_j} \cdot S_0})}{\beta} + d_{\text{metas}} \cdot n \cdot (t_{j-1} - t_j)}$$

m=1:

$$\mathbb{P}(D(t_j) = 0 | D(t_{j-1}) = 0, N(t_{j-1}) = n, N(t_j) = n + 1) =$$

$$\frac{\beta e^{\frac{2d_{\text{size}}\left(\sqrt{e^{\beta t_{j-1}}}-\sqrt{e^{\beta t_j}}\right)\sqrt{S_0}}{\beta}+d_{\text{metas}}nt_{j-1}-d_{\text{metas}}(1+n)t_j}}{d_{\text{metas}}(\beta+2d_{\text{metas}})\left(2\left(\sqrt{e^{\beta t_{j-1}}}-\sqrt{e^{\beta t_j}}\right)m_{\text{size}}\sqrt{S_0}+\beta m_{\text{basal}}(t_{j-1}-t_j)\right)}$$
$$\cdot\left(\beta\left(e^{d_{\text{metas}}t_{j-1}}-e^{d_{\text{metas}}t_j}\right)m_{\text{basal}}+2d_{\text{metas}}e^{d_{\text{metas}}t_{j-1}}\left(m_{\text{basal}}+m_{\text{size}}\sqrt{e^{\beta t_{j-1}}S_0}\right)\right.$$
$$\left.-2d_{\text{metas}}e^{d_{\text{metas}}t_j}\left(m_{\text{basal}}+m_{\text{size}}\sqrt{e^{\beta t_j}S_0}\right)\right)$$

m=2:

$$\mathbb{P}(D(t_j)=0|D(t_{j-1})=0,N(t_{j-1})=n,N(t_j)=n+2)=$$
$$\frac{\beta^2 e^{\frac{2d_{\text{size}}\left(\sqrt{e^{\beta t_{j-1}}}-\sqrt{e^{\beta t_j}}\right)\sqrt{S_0}}{\beta}+d_{\text{metas}}nt_{j-1}-d_{\text{metas}}(2+n)t_j}}{d_{\text{metas}}^2(\beta+2d_{\text{metas}})^2\left(2\left(\sqrt{e^{\beta t_{j-1}}}-\sqrt{e^{\beta t_j}}\right)m_{\text{size}}\sqrt{S_0}+\beta m_{\text{basal}}(t_{j-1}-t_j)\right)^2}$$
$$\cdot\left(\beta^2\left(e^{d_{\text{metas}}t_{j-1}}-e^{d_{\text{metas}}t_j}\right)^2 m_{\text{basal}}^2\right.$$
$$+4\beta d_{\text{metas}}\left(e^{d_{\text{metas}}t_{j-1}}-e^{d_{\text{metas}}t_j}\right)$$
$$\cdot m_{\text{basal}}\left(e^{d_{\text{metas}}t_{j-1}}\left(m_{\text{basal}}+m_{\text{size}}\sqrt{e^{\beta t_{j-1}}S_0}\right)-e^{d_{\text{metas}}t_j}\left(m_{\text{basal}}+m_{\text{size}}\sqrt{e^{\beta t_j}S_0}\right)\right)$$
$$+4d_{\text{metas}}^2\left[e^{(\beta+2d_{\text{metas}})t_{j-1}}m_{\text{size}}^2S_0+e^{(\beta+2d_{\text{metas}})t_j}m_{\text{size}}^2S_0\right.$$
$$+e^{2d_{\text{metas}}t_{j-1}}m_{\text{basal}}\left(m_{\text{basal}}+2m_{\text{size}}\sqrt{e^{\beta t_{j-1}}S_0}\right)$$
$$-2e^{d_{\text{metas}}(t_{j-1}+t_j)}\left(m_{\text{basal}}+m_{\text{size}}\sqrt{e^{\beta t_{j-1}}S_0}\right)\left(m_{\text{basal}}+m_{\text{size}}\sqrt{e^{\beta t_j}S_0}\right)$$
$$\left.\left.+e^{2d_{\text{metas}}t_j}m_{\text{basal}}\left(m_{\text{basal}}+2m_{\text{size}}\sqrt{e^{\beta t_j}S_0}\right)\right]\right)$$

m=3:

$$\mathbb{P}(D(t_j)=0|D(t_{j-1})=0,N(t_{j-1})=n,N(t_j)=n+3)=$$

$$\frac{\beta^3 e^{\frac{2d_{\text{size}}\left(\sqrt{e^{\beta t_{j-1}}}-\sqrt{e^{\beta t_j}}\right)\sqrt{S_0}}{\beta}+d_{\text{metas}}nt_{j-1}-d_{\text{metas}}(3+n)t_j}}{d_{\text{metas}}^3(\beta+2d_{\text{metas}})^3\left(2\left(-\sqrt{e^{\beta t_{j-1}}}+\sqrt{e^{\beta t_j}}\right)m_{\text{size}}\sqrt{S_0}+m_{\text{basal}}(-t_{j-1}+t_j)\right)^3}$$

$$\cdot\Bigg(-\beta^3\left(e^{d_{\text{metas}}t_{j-1}}-e^{d_{\text{metas}}t_j}\right)^3 m_{\text{basal}}^3$$

$$-6\beta^2 d_{\text{metas}}\left(e^{d_{\text{metas}}t_{j-1}}-e^{d_{\text{metas}}t_j}\right)^2 m_{\text{basal}}^2$$

$$\cdot\left(e^{d_{\text{metas}}t_{j-1}}\left(m_{\text{basal}}+m_{\text{size}}\sqrt{e^{\beta t_{j-1}}S_0}\right)-e^{d_{\text{metas}}t_j}\left(m_{\text{basal}}+m_{\text{size}}\sqrt{e^{\beta t_j}S_0}\right)\right)$$

$$-12\beta d_{\text{metas}}^2\left(e^{d_{\text{metas}}t_{j-1}}-e^{d_{\text{metas}}t_j}\right)m_{\text{basal}}$$

$$\cdot\Big(e^{(\beta+2d_{\text{metas}})t_{j-1}}m_{\text{size}}^2 S_0+e^{(\beta+2d_{\text{metas}})t_j}m_{\text{size}}^2 S_0$$

$$+e^{2d_{\text{metas}}t_{j-1}}m_{\text{basal}}\left(m_{\text{basal}}+2m_{\text{size}}\sqrt{e^{\beta t_{j-1}}S_0}\right)$$

$$-2e^{d_{\text{metas}}(t_{j-1}+t_j)}\left(m_{\text{basal}}+m_{\text{size}}\sqrt{e^{\beta t_{j-1}}S_0}\right)\left(m_{\text{basal}}+m_{\text{size}}\sqrt{e^{\beta t_j}S_0}\right)$$

$$+e^{2d_{\text{metas}}t_j}m_{\text{basal}}\left(m_{\text{basal}}+2m_{\text{size}}\sqrt{e^{\beta t_j}S_0}\right)\Big)$$

$$-8d_{\text{metas}}^3\Big[3e^{d_{\text{metas}}t_{j-1}+\beta t_j+2d_{\text{metas}}t_j}m_{\text{size}}^2 S_0\left(m_{\text{basal}}+m_{\text{size}}\sqrt{e^{\beta t_{j-1}}S_0}\right)$$

$$+e^{(\beta+3d_{\text{metas}})t_{j-1}}m_{\text{size}}^2 S_0\left(3m_{\text{basal}}+m_{\text{size}}\sqrt{e^{\beta t_{j-1}}S_0}\right)$$

$$+e^{3d_{\text{metas}}t_{j-1}}m_{\text{basal}}^2\left(m_{\text{basal}}+3m_{\text{size}}\sqrt{e^{\beta t_{j-1}}S_0}\right)$$

$$-3e^{\beta t_{j-1}+2d_{\text{metas}}t_{j-1}+d_{\text{metas}}t_j}m_{\text{size}}^2 S_0\left(m_{\text{basal}}+m_{\text{size}}\sqrt{e^{\beta t_j}S_0}\right)$$

$$-3e^{d_{\text{metas}}(2t_{j-1}+t_j)}m_{\text{basal}}\left(m_{\text{basal}}+2m_{\text{size}}\sqrt{e^{\beta t_{j-1}}S_0}\right)\left(m_{\text{basal}}+m_{\text{size}}\sqrt{e^{\beta t_j}S_0}\right)$$

$$-e^{(\beta+3d_{\text{metas}})t_j}m_{\text{size}}^2 S_0\left(3m_{\text{basal}}+m_{\text{size}}\sqrt{e^{\beta t_j}S_0}\right)$$

$$+3e^{d_{\text{metas}}(t_{j-1}+2t_j)}m_{\text{basal}}\left(m_{\text{basal}}+m_{\text{size}}\sqrt{e^{\beta t_{j-1}}S_0}\right)\left(m_{\text{basal}}+2m_{\text{size}}\sqrt{e^{\beta t_j}S_0}\right)$$

$$-e^{3d_{\text{metas}}t_j}m_{\text{basal}}^2\left(m_{\text{basal}}+3m_{\text{size}}\sqrt{e^{\beta t_j}S_0}\right)\Big]\Bigg)$$

m=4:

$$\mathbb{P}(D(t_j)=0|D(t_{j-1})=0,N(t_{j-1})=n,N(t_j)=n+4)=$$

$$\frac{\beta^4 e^{d_{\text{metas}} n t_{j-1} - d_{\text{metas}}(n+4)t_j + \frac{2 d_{\text{size}}\left(\sqrt{e^{\beta t_{j-1}}} - \sqrt{e^{\beta t_j}}\right)\sqrt{S_0}}{\beta}}}{d_{\text{metas}}^4 (\beta + 2 d_{\text{metas}})^4 \left(\beta m_{\text{basal}}(t_{j-1} - t_j) + 2 m_{\text{size}}\left(\sqrt{e^{\beta t_{j-1}}} - \sqrt{e^{\beta t_j}}\right)\sqrt{S_0}\right)^4}$$

$$\cdot \Bigg(16 \Big[-4 e^{d_{\text{metas}}(t_{j-1}+3t_j)} m_{\text{size}}^3 \left(m_{\text{basal}} + m_{\text{size}}\sqrt{e^{\beta t_{j-1}} S_0}\right)\left(e^{\beta t_j} S_0\right)^{3/2}$$

$$+ e^{2(\beta+2d_{\text{metas}})t_{j-1}} m_{\text{size}}^4 S_0^2 + e^{2(\beta+2d_{\text{metas}})t_j} m_{\text{size}}^4 S_0^2 + 6 e^{(\beta+2d_{\text{metas}})(t_{j-1}+t_j)} m_{\text{size}}^4 S_0^2$$

$$- 12 e^{d_{\text{metas}} t_{j-1} + \beta t_j + 3 d_{\text{metas}} t_j} m_{\text{basal}} m_{\text{size}}^2 S_0 \left(m_{\text{basal}} + m_{\text{size}}\sqrt{e^{\beta t_{j-1}} S_0}\right)$$

$$+ 6 e^{\beta t_j + 2 d_{\text{metas}}(t_{j-1}+t_j)} m_{\text{basal}} m_{\text{size}}^2 S_0 \left(m_{\text{basal}} + 2 m_{\text{size}}\sqrt{e^{\beta t_{j-1}} S_0}\right)$$

$$+ 2 e^{(\beta+4d_{\text{metas}})t_{j-1}} m_{\text{basal}} m_{\text{size}}^2 S_0 \left(3 m_{\text{basal}} + 2 m_{\text{size}}\sqrt{e^{\beta t_{j-1}} S_0}\right)$$

$$+ e^{4 d_{\text{metas}} t_{j-1}} m_{\text{basal}}^3 \left(m_{\text{basal}} + 4 m_{\text{size}}\sqrt{e^{\beta t_{j-1}} S_0}\right)$$

$$- 4 e^{\beta t_{j-1} + 3 d_{\text{metas}} t_{j-1} + d_{\text{metas}} t_j} m_{\text{size}}^2 S_0 \left(3 m_{\text{basal}} + m_{\text{size}}\sqrt{e^{\beta t_{j-1}} S_0}\right)$$

$$\cdot \left(m_{\text{basal}} + m_{\text{size}}\sqrt{e^{\beta t_j} S_0}\right)$$

$$- 4 e^{d_{\text{metas}}(3t_{j-1}+t_j)} m_{\text{basal}}^2 \left(m_{\text{basal}} + 3 m_{\text{size}}\sqrt{e^{\beta t_{j-1}} S_0}\right)\left(m_{\text{basal}} + m_{\text{size}}\sqrt{e^{\beta t_j} S_0}\right)$$

$$+ 6 e^{\beta t_{j-1} + 2 d_{\text{metas}}(t_{j-1}+t_j)} m_{\text{basal}} m_{\text{size}}^2 S_0 \left(m_{\text{basal}} + 2 m_{\text{size}}\sqrt{e^{\beta t_j} S_0}\right)$$

$$+ 6 e^{2 d_{\text{metas}}(t_{j-1}+t_j)} m_{\text{basal}}^2 \left(m_{\text{basal}} + 2 m_{\text{size}}\sqrt{e^{\beta t_{j-1}} S_0}\right)\left(m_{\text{basal}} + 2 m_{\text{size}}\sqrt{e^{\beta t_j} S_0}\right)$$

$$+ 2 e^{(\beta+4d_{\text{metas}})t_j} m_{\text{basal}} m_{\text{size}}^2 S_0 \left(3 m_{\text{basal}} + 2 m_{\text{size}}\sqrt{e^{\beta t_j} S_0}\right)$$

$$- 4 e^{d_{\text{metas}}(t_{j-1}+3t_j)} m_{\text{basal}}^2 \left(m_{\text{basal}} + m_{\text{size}}\sqrt{e^{\beta t_{j-1}} S_0}\right)\left(m_{\text{basal}} + 3 m_{\text{size}}\sqrt{e^{\beta t_j} S_0}\right)$$

$$+ e^{4 d_{\text{metas}} t_j} m_{\text{basal}}^3 \left(m_{\text{basal}} + 4 m_{\text{size}}\sqrt{e^{\beta t_j} S_0}\right)\Big] d_{\text{metas}}^4$$

$$+ 32 \beta m_{\text{basal}} \Big[-e^{d_{\text{metas}}(3t_{j-1}+t_j)} m_{\text{size}}^3 \left(e^{\beta t_{j-1}} S_0\right)^{3/2}$$

$$+ 3 e^{\beta t_j + 2 d_{\text{metas}}(t_{j-1}+t_j)} m_{\text{size}}^2 S_0 \left(m_{\text{basal}} + m_{\text{size}}\sqrt{e^{\beta t_{j-1}} S_0}\right)$$

$$+ e^{(\beta+4d_{\text{metas}})t_{j-1}} m_{\text{size}}^2 S_0 \left(3 m_{\text{basal}} + m_{\text{size}}\sqrt{e^{\beta t_{j-1}} S_0}\right)$$

$$+ e^{4 d_{\text{metas}} t_{j-1}} m_{\text{basal}}^2 \left(m_{\text{basal}} + 3 m_{\text{size}}\sqrt{e^{\beta t_{j-1}} S_0}\right)$$

$$+ 3 e^{\beta t_{j-1} + 2 d_{\text{metas}}(t_{j-1}+t_j)} m_{\text{size}}^2 S_0 \left(m_{\text{basal}} + m_{\text{size}}\sqrt{e^{\beta t_j} S_0}\right)$$

$$- 3 e^{\beta t_{j-1} + 3 d_{\text{metas}} t_{j-1} + d_{\text{metas}} t_j} m_{\text{size}}^2 S_0 \left(2 m_{\text{basal}} + m_{\text{size}}\sqrt{e^{\beta t_j} S_0}\right)$$

$$+ e^{(\beta+4d_{\text{metas}})t_j} m_{\text{size}}^2 S_0 \left(3 m_{\text{basal}} + m_{\text{size}}\sqrt{e^{\beta t_j} S_0}\right)$$

$$+ e^{4 d_{\text{metas}} t_j} m_{\text{basal}}^2 \left(m_{\text{basal}} + 3 m_{\text{size}}\sqrt{e^{\beta t_j} S_0}\right)$$

$$
\begin{aligned}
&+ 3e^{2d_{\text{metas}}(t_{j-1}+t_j)} m_{\text{basal}} \left(2m_{\text{basal}}^2 + 3m_{\text{size}} \left(\sqrt{e^{\beta t_{j-1}}S_0} + \sqrt{e^{\beta t_j}S_0}\right) m_{\text{basal}} \right.\\
&\quad \left. +4m_{\text{size}}^2 \sqrt{e^{\beta t_{j-1}}S_0}\sqrt{e^{\beta t_j}S_0}\right)\\
&- e^{\beta t_j + d_{\text{metas}}(t_{j-1}+3t_j)} m_{\text{size}}^2 S_0 \left(6m_{\text{basal}} + m_{\text{size}} \left(3\sqrt{e^{\beta t_{j-1}}S_0} + \sqrt{e^{\beta t_j}S_0}\right)\right)\\
&- e^{d_{\text{metas}}(3t_{j-1}+t_j)} m_{\text{basal}} \left(4m_{\text{basal}}^2 + 3m_{\text{size}} \left(3\sqrt{e^{\beta t_{j-1}}S_0} + \sqrt{e^{\beta t_j}S_0}\right) m_{\text{basal}} \right.\\
&\quad \left. +6m_{\text{size}}^2 \sqrt{e^{\beta t_{j-1}}S_0}\sqrt{e^{\beta t_j}S_0}\right)\\
&- e^{d_{\text{metas}}(t_{j-1}+3t_j)} m_{\text{basal}} \left(4m_{\text{basal}}^2 + 3m_{\text{size}} \left(\sqrt{e^{\beta t_{j-1}}S_0} + 3\sqrt{e^{\beta t_j}S_0}\right) m_{\text{basal}} \right.\\
&\quad \left. \left. +6m_{\text{size}}^2 \sqrt{e^{\beta t_{j-1}}S_0}\sqrt{e^{\beta t_j}S_0}\right)\right] d_{\text{metas}}^3\\
&+ 24\beta^2 m_{\text{basal}}^2 d_{\text{metas}}^2 \left[e^{(\beta+4d_{\text{metas}})t_{j-1}} S_0 m_{\text{size}}^2 + e^{(\beta+4d_{\text{metas}})t_j} S_0 m_{\text{size}}^2 \right.\\
&\quad -2e^{\beta t_{j-1}+3d_{\text{metas}}t_{j-1}+d_{\text{metas}}t_j} S_0 m_{\text{size}}^2 - 2e^{d_{\text{metas}}t_{j-1}+\beta t_j+3d_{\text{metas}}t_j} S_0 m_{\text{size}}^2\\
&\quad + e^{\beta t_{j-1}+2d_{\text{metas}}(t_{j-1}+t_j)} S_0 m_{\text{size}}^2 + e^{\beta t_j+2d_{\text{metas}}(t_{j-1}+t_j)} S_0 m_{\text{size}}^2\\
&\quad + e^{4d_{\text{metas}}t_{j-1}} m_{\text{basal}} \left(m_{\text{basal}} + 2m_{\text{size}}\sqrt{e^{\beta t_{j-1}}S_0}\right)\\
&\quad + e^{4d_{\text{metas}}t_j} m_{\text{basal}} \left(m_{\text{basal}} + 2m_{\text{size}}\sqrt{e^{\beta t_j}S_0}\right)\\
&\quad + e^{2d_{\text{metas}}(t_{j-1}+t_j)} \left(6m_{\text{basal}}^2 + 6m_{\text{size}} \left(\sqrt{e^{\beta t_{j-1}}S_0} + \sqrt{e^{\beta t_j}S_0}\right) m_{\text{basal}} \right.\\
&\qquad \left. +4m_{\text{size}}^2 \sqrt{e^{\beta t_{j-1}}S_0}\sqrt{e^{\beta t_j}S_0}\right)\\
&\quad - 2e^{d_{\text{metas}}(3t_{j-1}+t_j)} \left(2m_{\text{basal}}^2 + m_{\text{size}} \left(3\sqrt{e^{\beta t_{j-1}}S_0} + \sqrt{e^{\beta t_j}S_0}\right) m_{\text{basal}} \right.\\
&\qquad \left. +m_{\text{size}}^2 \sqrt{e^{\beta t_{j-1}}S_0}\sqrt{e^{\beta t_j}S_0}\right)\\
&\quad - 2e^{d_{\text{metas}}(t_{j-1}+3t_j)} \left(2m_{\text{basal}}^2 + m_{\text{size}} \left(\sqrt{e^{\beta t_{j-1}}S_0} + 3\sqrt{e^{\beta t_j}S_0}\right) m_{\text{basal}} \right.\\
&\qquad \left. \left. +m_{\text{size}}^2 \sqrt{e^{\beta t_{j-1}}S_0}\sqrt{e^{\beta t_j}S_0}\right)\right]\\
&+ 8\beta^3 \left(e^{d_{\text{metas}}t_{j-1}} - e^{d_{\text{metas}}t_j}\right)^3 m_{\text{basal}}^3 d_{\text{metas}}\\
&\quad \cdot \left[e^{d_{\text{metas}}t_{j-1}} \left(m_{\text{basal}} + m_{\text{size}}\sqrt{e^{\beta t_{j-1}}S_0}\right) - e^{d_{\text{metas}}t_j} \left(m_{\text{basal}} + m_{\text{size}}\sqrt{e^{\beta t_j}S_0}\right)\right]\\
&\left. + \beta^4 \left(e^{d_{\text{metas}}t_{j-1}} - e^{d_{\text{metas}}t_j}\right)^4 m_{\text{basal}}^4 \right)
\end{aligned}
$$

m=5:

$$\mathbb{P}(D(t_j) = 0 | D(t_{j-1}) = 0, N(t_{j-1}) = n, N(t_j) = n+5) =$$

$$
-\frac{\beta^5 e^{d_{\text{metas}} n(t_{j-1}-t_j)-5d_{\text{metas}}t_j+\frac{2d_{\text{size}}\left(\sqrt{e^{\beta t_{j-1}}}-\sqrt{e^{\beta t_j}}\right)\sqrt{S_0}}{\beta}}}{d_{\text{metas}}^5(\beta+2d_{\text{metas}})^5\left(\beta m_{\text{basal}}(t_{j-1}-t_j)+2m_{\text{size}}\left(\sqrt{e^{\beta t_{j-1}}}-\sqrt{e^{\beta t_j}}\right)\sqrt{S_0}\right)^5}
$$

$$
\cdot\Bigg(-32d_{\text{metas}}^5\Big[e^{5d_{\text{metas}}t_{j-1}}\left(m_{\text{basal}}+5m_{\text{size}}\sqrt{e^{\beta t_{j-1}}S_0}\right)m_{\text{basal}}^4
$$

$$
-e^{5d_{\text{metas}}t_j}\left(m_{\text{basal}}+5m_{\text{size}}\sqrt{e^{\beta t_j}S_0}\right)m_{\text{basal}}^4\Big]
$$

$$
-5e^{d_{\text{metas}}(4t_{j-1}+t_j)}\left(m_{\text{basal}}+4m_{\text{size}}\sqrt{e^{\beta t_{j-1}}S_0}\right)\left(m_{\text{basal}}+m_{\text{size}}\sqrt{e^{\beta t_j}S_0}\right)m_{\text{basal}}^3
$$

$$
+10e^{3d_{\text{metas}}t_{j-1}+2d_{\text{metas}}t_j}\left(m_{\text{basal}}+3m_{\text{size}}\sqrt{e^{\beta t_{j-1}}S_0}\right)\left(m_{\text{basal}}+2m_{\text{size}}\sqrt{e^{\beta t_j}S_0}\right)m_{\text{basal}}^3
$$

$$
-10e^{2d_{\text{metas}}t_{j-1}+3d_{\text{metas}}t_j}\left(m_{\text{basal}}+2m_{\text{size}}\sqrt{e^{\beta t_{j-1}}S_0}\right)\left(m_{\text{basal}}+3m_{\text{size}}\sqrt{e^{\beta t_j}S_0}\right)m_{\text{basal}}^3
$$

$$
+5e^{d_{\text{metas}}(t_{j-1}+4t_j)}\left(m_{\text{basal}}+m_{\text{size}}\sqrt{e^{\beta t_{j-1}}S_0}\right)\left(m_{\text{basal}}+4m_{\text{size}}\sqrt{e^{\beta t_j}S_0}\right)m_{\text{basal}}^3
$$

$$
+10e^{(\beta+5d_{\text{metas}})t_{j-1}}m_{\text{size}}^2S_0\left(m_{\text{basal}}+m_{\text{size}}\sqrt{e^{\beta t_{j-1}}S_0}\right)m_{\text{basal}}^2
$$

$$
+10e^{3d_{\text{metas}}t_{j-1}+\beta t_j+2d_{\text{metas}}t_j}m_{\text{size}}^2S_0\left(m_{\text{basal}}+3m_{\text{size}}\sqrt{e^{\beta t_{j-1}}S_0}\right)m_{\text{basal}}^2
$$

$$
-10e^{(\beta+5d_{\text{metas}})t_j}m_{\text{size}}^2S_0\left(m_{\text{basal}}+m_{\text{size}}\sqrt{e^{\beta t_j}S_0}\right)m_{\text{basal}}^2
$$

$$
-10e^{\beta t_{j-1}+2d_{\text{metas}}t_{j-1}+3d_{\text{metas}}t_j}m_{\text{size}}^2S_0\left(m_{\text{basal}}+3m_{\text{size}}\sqrt{e^{\beta t_j}S_0}\right)m_{\text{basal}}^2
$$

$$
-10e^{\beta t_{j-1}+d_{\text{metas}}(4t_{j-1}+t_j)}m_{\text{size}}^2S_0\left(3m_{\text{basal}}+2m_{\text{size}}\sqrt{e^{\beta t_{j-1}}S_0}\right)
$$

$$
\cdot\left(m_{\text{basal}}+m_{\text{size}}\sqrt{e^{\beta t_j}S_0}\right)m_{\text{basal}}
$$

$$
-10e^{2d_{\text{metas}}t_{j-1}+\beta t_j+3d_{\text{metas}}t_j}m_{\text{size}}^2S_0\left(m_{\text{basal}}+2m_{\text{size}}\sqrt{e^{\beta t_{j-1}}S_0}\right)
$$

$$
\cdot\left(3m_{\text{basal}}+m_{\text{size}}\sqrt{e^{\beta t_j}S_0}\right)m_{\text{basal}}
$$

$$
+10e^{\beta t_{j-1}+3d_{\text{metas}}t_{j-1}+2d_{\text{metas}}t_j}m_{\text{size}}^2S_0\left(3m_{\text{basal}}+m_{\text{size}}\sqrt{e^{\beta t_{j-1}}S_0}\right)
$$

$$
\cdot\left(m_{\text{basal}}+2m_{\text{size}}\sqrt{e^{\beta t_j}S_0}\right)m_{\text{basal}}
$$

$$
+10e^{\beta t_j+d_{\text{metas}}(t_{j-1}+4t_j)}m_{\text{size}}^2S_0\left(m_{\text{basal}}+m_{\text{size}}\sqrt{e^{\beta t_{j-1}}S_0}\right)
$$

$$
\cdot\left(3m_{\text{basal}}+2m_{\text{size}}\sqrt{e^{\beta t_j}S_0}\right)m_{\text{basal}}
$$

$$
+5e^{d_{\text{metas}}t_{j-1}+2\beta t_j+4d_{\text{metas}}t_j}m_{\text{size}}^4S_0^2\left(m_{\text{basal}}+m_{\text{size}}\sqrt{e^{\beta t_{j-1}}S_0}\right)
$$

$$
+10e^{\beta t_{j-1}+3d_{\text{metas}}t_{j-1}+\beta t_j+2d_{\text{metas}}t_j}m_{\text{size}}^4S_0^2\left(3m_{\text{basal}}+m_{\text{size}}\sqrt{e^{\beta t_{j-1}}S_0}\right)
$$

$$
+e^{(2\beta+5d_{\text{metas}})t_{j-1}}m_{\text{size}}^4S_0^2\left(5m_{\text{basal}}+m_{\text{size}}\sqrt{e^{\beta t_{j-1}}S_0}\right)
$$

$$
-5e^{2\beta t_{j-1}+4d_{\text{metas}}t_{j-1}+d_{\text{metas}}t_j}m_{\text{size}}^4S_0^2\left(m_{\text{basal}}+m_{\text{size}}\sqrt{e^{\beta t_j}S_0}\right)
$$

$$
\begin{aligned}
&- 10e^{\beta t_{j-1}+2d_{\text{metas}}t_{j-1}+\beta t_j+3d_{\text{metas}}t_j} m_{\text{size}}^4 S_0^2 \left(3m_{\text{basal}} + m_{\text{size}}\sqrt{e^{\beta t_j}S_0}\right) \\
&-e^{(2\beta+5d_{\text{metas}})t_j} m_{\text{size}}^4 S_0^2 \left(5m_{\text{basal}} + m_{\text{size}}\sqrt{e^{\beta t_j}S_0}\right)\Big] \\
- 80\beta m_{\text{basal}} d_{\text{metas}}^4 &\Big[-4e^{d_{\text{metas}}(4t_{j-1}+t_j)} m_{\text{size}}^3 \left(2m_{\text{basal}} + m_{\text{size}}\sqrt{e^{\beta t_j}S_0}\right)\left(e^{\beta t_{j-1}}S_0\right)^{3/2} \\
&+ e^{(2\beta+5d_{\text{metas}})t_{j-1}} m_{\text{size}}^4 S_0^2 - e^{(2\beta+5d_{\text{metas}})t_j} m_{\text{size}}^4 S_0^2 - e^{2\beta t_{j-1}+4d_{\text{metas}}t_{j-1}+d_{\text{metas}}t_j} m_{\text{size}}^4 S_0^2 \\
&+ 6e^{\beta t_{j-1}+3d_{\text{metas}}t_{j-1}+\beta t_j+2d_{\text{metas}}t_j} m_{\text{size}}^4 S_0^2 - 6e^{\beta t_{j-1}+2d_{\text{metas}}t_{j-1}+\beta t_j+3d_{\text{metas}}t_j} m_{\text{size}}^4 S_0^2 \\
&+ e^{d_{\text{metas}}t_{j-1}+2\beta t_j+4d_{\text{metas}}t_j} m_{\text{size}}^4 S_0^2 + 4e^{d_{\text{metas}}(t_{j-1}+4t_j)} m_{\text{size}}^3 \left(e^{\beta t_j}S_0\right)^{3/2} \\
&\quad \cdot \left(2m_{\text{basal}} + m_{\text{size}}\sqrt{e^{\beta t_{j-1}}S_0}\right) \\
&+ 6e^{3d_{\text{metas}}t_{j-1}+\beta t_j+2d_{\text{metas}}t_j} m_{\text{basal}} m_{\text{size}}^2 S_0 \left(m_{\text{basal}} + 2m_{\text{size}}\sqrt{e^{\beta t_{j-1}}S_0}\right) \\
&+ 2e^{(\beta+5d_{\text{metas}})t_{j-1}} m_{\text{basal}} m_{\text{size}}^2 S_0 \left(3m_{\text{basal}} + 2m_{\text{size}}\sqrt{e^{\beta t_{j-1}}S_0}\right) \\
&+ 6e^{\beta t_j+d_{\text{metas}}(t_{j-1}+4t_j)} m_{\text{basal}} m_{\text{size}}^2 S_0 \left(3m_{\text{basal}} + 2m_{\text{size}}\sqrt{e^{\beta t_{j-1}}S_0}\right) \\
&+ e^{5d_{\text{metas}}t_{j-1}} m_{\text{basal}}^3 \left(m_{\text{basal}} + 4m_{\text{size}}\sqrt{e^{\beta t_{j-1}}S_0}\right) \\
&- 6e^{\beta t_{j-1}+2d_{\text{metas}}t_{j-1}+3d_{\text{metas}}t_j} m_{\text{basal}} m_{\text{size}}^2 S_0 \left(m_{\text{basal}} + 2m_{\text{size}}\sqrt{e^{\beta t_j}S_0}\right) \\
&- 2e^{(\beta+5d_{\text{metas}})t_j} m_{\text{basal}} m_{\text{size}}^2 S_0 \left(3m_{\text{basal}} + 2m_{\text{size}}\sqrt{e^{\beta t_j}S_0}\right) \\
&- 6e^{\beta t_{j-1}+d_{\text{metas}}(4t_{j-1}+t_j)} m_{\text{basal}} m_{\text{size}}^2 S_0 \left(3m_{\text{basal}} + 2m_{\text{size}}\sqrt{e^{\beta t_j}S_0}\right) \\
&- e^{5d_{\text{metas}}t_j} m_{\text{basal}}^3 \left(m_{\text{basal}} + 4m_{\text{size}}\sqrt{e^{\beta t_j}S_0}\right) \\
&- e^{d_{\text{metas}}(4t_{j-1}+t_j)} m_{\text{basal}}^2 \left(5m_{\text{basal}}^2 + 4m_{\text{size}}\left(4\sqrt{e^{\beta t_{j-1}}S_0} + \sqrt{e^{\beta t_j}S_0}\right) m_{\text{basal}}\right. \\
&\quad \left. +12m_{\text{size}}^2 \sqrt{e^{\beta t_{j-1}}S_0}\sqrt{e^{\beta t_j}S_0}\right) \\
&- 2e^{2d_{\text{metas}}t_{j-1}+\beta t_j+3d_{\text{metas}}t_j} m_{\text{size}}^2 S_0 \left(9m_{\text{basal}}^2 + 2m_{\text{size}}\left(6\sqrt{e^{\beta t_{j-1}}S_0} + \sqrt{e^{\beta t_j}S_0}\right) m_{\text{basal}}\right. \\
&\quad \left. +2m_{\text{size}}^2 \sqrt{e^{\beta t_{j-1}}S_0}\sqrt{e^{\beta t_j}S_0}\right) \\
&+ 2e^{3d_{\text{metas}}t_{j-1}+2d_{\text{metas}}t_j} m_{\text{basal}}^2 \left(5m_{\text{basal}}^2 + 4m_{\text{size}}\left(3\sqrt{e^{\beta t_{j-1}}S_0} + 2\sqrt{e^{\beta t_j}S_0}\right) m_{\text{basal}}\right. \\
&\quad \left. +18m_{\text{size}}^2 \sqrt{e^{\beta t_{j-1}}S_0}\sqrt{e^{\beta t_j}S_0}\right) \\
&- 2e^{2d_{\text{metas}}t_{j-1}+3d_{\text{metas}}t_j} m_{\text{basal}}^2 \left(5m_{\text{basal}}^2 + 4m_{\text{size}}\left(2\sqrt{e^{\beta t_{j-1}}S_0} + 3\sqrt{e^{\beta t_j}S_0}\right) m_{\text{basal}}\right. \\
&\quad \left. +18m_{\text{size}}^2 \sqrt{e^{\beta t_{j-1}}S_0}\sqrt{e^{\beta t_j}S_0}\right) \\
&+ e^{d_{\text{metas}}(t_{j-1}+4t_j)} m_{\text{basal}}^2 \left(5m_{\text{basal}}^2 + 4m_{\text{size}}\left(\sqrt{e^{\beta t_{j-1}}S_0} + 4\sqrt{e^{\beta t_j}S_0}\right) m_{\text{basal}}\right. \\
&\quad \left. +12m_{\text{size}}^2 \sqrt{e^{\beta t_{j-1}}S_0}\sqrt{e^{\beta t_j}S_0}\right)
\end{aligned}
$$

$$
\begin{aligned}
&+ 2e^{\beta t_{j-1}+3d_{\mathrm{metas}}t_{j-1}+2d_{\mathrm{metas}}t_j} m_{\mathrm{size}}^2 S_0 \left(9m_{\mathrm{basal}}^2 + 2m_{\mathrm{size}}\left(\sqrt{e^{\beta t_{j-1}}S_0} + 6\sqrt{e^{\beta t_j}S_0}\right) m_{\mathrm{basal}}\right.\\
&\quad \left.+2m_{\mathrm{size}}^2\sqrt{e^{\beta t_{j-1}}S_0}\sqrt{e^{\beta t_j}S_0}\right)\Bigg]\\
&- 80\beta^2 m_{\mathrm{basal}}^2 d_{\mathrm{metas}}^3 \Bigg[e^{5d_{\mathrm{metas}}t_{j-1}}\left(m_{\mathrm{basal}} + 3m_{\mathrm{size}}\sqrt{e^{\beta t_{j-1}}S_0}\right) m_{\mathrm{basal}}^2\\
&- e^{5d_{\mathrm{metas}}t_j}\left(m_{\mathrm{basal}} + 3m_{\mathrm{size}}\sqrt{e^{\beta t_j}S_0}\right) m_{\mathrm{basal}}^2\\
&+ 2e^{3d_{\mathrm{metas}}t_{j-1}+2d_{\mathrm{metas}}t_j}\left(5m_{\mathrm{basal}}^2 + 9m_{\mathrm{size}}\sqrt{e^{\beta t_{j-1}}S_0}m_{\mathrm{basal}} + 6m_{\mathrm{size}}\sqrt{e^{\beta t_j}S_0}m_{\mathrm{basal}}\right.\\
&\quad \left.+9m_{\mathrm{size}}^2\sqrt{e^{\beta t_{j-1}}S_0}\sqrt{e^{\beta t_j}S_0}\right) m_{\mathrm{basal}}\\
&- 2e^{2d_{\mathrm{metas}}t_{j-1}+3d_{\mathrm{metas}}t_j}\left(5m_{\mathrm{basal}}^2 + 6m_{\mathrm{size}}\sqrt{e^{\beta t_{j-1}}S_0}m_{\mathrm{basal}} + 9m_{\mathrm{size}}\sqrt{e^{\beta t_j}S_0}m_{\mathrm{basal}}\right.\\
&\quad \left.+9m_{\mathrm{size}}^2\sqrt{e^{\beta t_{j-1}}S_0}\sqrt{e^{\beta t_j}S_0}\right) m_{\mathrm{basal}}\\
&- e^{d_{\mathrm{metas}}(4t_{j-1}+t_j)}\left(5m_{\mathrm{basal}}^2 + 3m_{\mathrm{size}}\left(4\sqrt{e^{\beta t_{j-1}}S_0} + \sqrt{e^{\beta t_j}S_0}\right) m_{\mathrm{basal}}\right.\\
&\quad \left.+6m_{\mathrm{size}}^2\sqrt{e^{\beta t_{j-1}}S_0}\sqrt{e^{\beta t_j}S_0}\right) m_{\mathrm{basal}}\\
&+ e^{d_{\mathrm{metas}}(t_{j-1}+4t_j)}\left(5m_{\mathrm{basal}}^2 + 3m_{\mathrm{size}}\left(\sqrt{e^{\beta t_{j-1}}S_0} + 4\sqrt{e^{\beta t_j}S_0}\right) m_{\mathrm{basal}}\right.\\
&\quad \left.+6m_{\mathrm{size}}^2\sqrt{e^{\beta t_{j-1}}S_0}\sqrt{e^{\beta t_j}S_0}\right) m_{\mathrm{basal}}\\
&+ 3e^{3d_{\mathrm{metas}}t_{j-1}+\beta t_j+2d_{\mathrm{metas}}t_j} m_{\mathrm{size}}^2 S_0\left(m_{\mathrm{basal}} + m_{\mathrm{size}}\sqrt{e^{\beta t_{j-1}}S_0}\right)\\
&+ e^{(\beta+5d_{\mathrm{metas}})t_{j-1}} m_{\mathrm{size}}^2 S_0\left(3m_{\mathrm{basal}} + m_{\mathrm{size}}\sqrt{e^{\beta t_{j-1}}S_0}\right)\\
&- 3e^{\beta t_{j-1}+2d_{\mathrm{metas}}t_{j-1}+3d_{\mathrm{metas}}t_j} m_{\mathrm{size}}^2 S_0\left(m_{\mathrm{basal}} + m_{\mathrm{size}}\sqrt{e^{\beta t_j}S_0}\right)\\
&- e^{(\beta+5d_{\mathrm{metas}})t_j} m_{\mathrm{size}}^2 S_0\left(3m_{\mathrm{basal}} + m_{\mathrm{size}}\sqrt{e^{\beta t_j}S_0}\right)\\
&+ e^{\beta t_j+d_{\mathrm{metas}}(t_{j-1}+4t_j)} m_{\mathrm{size}}^2 S_0\left(9m_{\mathrm{basal}} + 3m_{\mathrm{size}}\sqrt{e^{\beta t_{j-1}}S_0} + 2m_{\mathrm{size}}\sqrt{e^{\beta t_j}S_0}\right)\\
&- e^{\beta t_{j-1}+d_{\mathrm{metas}}(4t_{j-1}+t_j)} m_{\mathrm{size}}^2 S_0\left(9m_{\mathrm{basal}} + 2m_{\mathrm{size}}\sqrt{e^{\beta t_{j-1}}S_0} + 3m_{\mathrm{size}}\sqrt{e^{\beta t_j}S_0}\right)\\
&- e^{2d_{\mathrm{metas}}t_{j-1}+\beta t_j+3d_{\mathrm{metas}}t_j} m_{\mathrm{size}}^2 S_0\left(9m_{\mathrm{basal}} + m_{\mathrm{size}}\left(6\sqrt{e^{\beta t_{j-1}}S_0} + \sqrt{e^{\beta t_j}S_0}\right)\right)\\
&+e^{\beta t_{j-1}+3d_{\mathrm{metas}}t_{j-1}+2d_{\mathrm{metas}}t_j} m_{\mathrm{size}}^2 S_0\left(9m_{\mathrm{basal}} + m_{\mathrm{size}}\left(\sqrt{e^{\beta t_{j-1}}S_0} + 6\sqrt{e^{\beta t_j}S_0}\right)\right)\Bigg]\\
&- 40\beta^3 m_{\mathrm{basal}}^3 d_{\mathrm{metas}}^2 \Bigg[e^{(\beta+5d_{\mathrm{metas}})t_{j-1}} S_0 m_{\mathrm{size}}^2 - e^{(\beta+5d_{\mathrm{metas}})t_j} S_0 m_{\mathrm{size}}^2\\
&-3e^{\beta t_{j-1}+4d_{\mathrm{metas}}t_{j-1}+d_{\mathrm{metas}}t_j} S_0 m_{\mathrm{size}}^2\\
&+ 3e^{\beta t_{j-1}+3d_{\mathrm{metas}}t_{j-1}+2d_{\mathrm{metas}}t_j} S_0 m_{\mathrm{size}}^2 + e^{3d_{\mathrm{metas}}t_{j-1}+\beta t_j+2d_{\mathrm{metas}}t_j} S_0 m_{\mathrm{size}}^2\\
&- e^{\beta t_{j-1}+2d_{\mathrm{metas}}t_{j-1}+3d_{\mathrm{metas}}t_j} S_0 m_{\mathrm{size}}^2 - 3e^{2d_{\mathrm{metas}}t_{j-1}+\beta t_j+3d_{\mathrm{metas}}t_j} S_0 m_{\mathrm{size}}^2\\
&+ 3e^{\beta t_j+d_{\mathrm{metas}}(t_{j-1}+4t_j)} S_0 m_{\mathrm{size}}^2\\
&+ e^{5d_{\mathrm{metas}}t_{j-1}} m_{\mathrm{basal}}\left(m_{\mathrm{basal}} + 2m_{\mathrm{size}}\sqrt{e^{\beta t_{j-1}}S_0}\right) - e^{5d_{\mathrm{metas}}t_j} m_{\mathrm{basal}}\left(m_{\mathrm{basal}} + 2m_{\mathrm{size}}\sqrt{e^{\beta t_j}S_0}\right)
\end{aligned}
$$

$$
\begin{aligned}
&+ 2e^{3d_{\text{metas}}t_{j-1}+2d_{\text{metas}}t_j} \left(5m_{\text{basal}}^2 + 6m_{\text{size}}\sqrt{e^{\beta t_{j-1}}S_0}m_{\text{basal}} + 4m_{\text{size}}\sqrt{e^{\beta t_j}S_0}m_{\text{basal}}\right.\\
&\quad\left.+3m_{\text{size}}^2\sqrt{e^{\beta t_{j-1}}S_0}\sqrt{e^{\beta t_j}S_0}\right)\\
&- 2e^{2d_{\text{metas}}t_{j-1}+3d_{\text{metas}}t_j} \left(5m_{\text{basal}}^2 + 4m_{\text{size}}\sqrt{e^{\beta t_{j-1}}S_0}m_{\text{basal}} + 6m_{\text{size}}\sqrt{e^{\beta t_j}S_0}m_{\text{basal}}\right.\\
&\quad\left.+3m_{\text{size}}^2\sqrt{e^{\beta t_{j-1}}S_0}\sqrt{e^{\beta t_j}S_0}\right)\\
&- e^{d_{\text{metas}}(4t_{j-1}+t_j)} \left(5m_{\text{basal}}^2 + 2m_{\text{size}}\left(4\sqrt{e^{\beta t_{j-1}}S_0} + \sqrt{e^{\beta t_j}S_0}\right)m_{\text{basal}}\right.\\
&\quad\left.+2m_{\text{size}}^2\sqrt{e^{\beta t_{j-1}}S_0}\sqrt{e^{\beta t_j}S_0}\right)\\
&+e^{d_{\text{metas}}(t_{j-1}+4t_j)} \left(5m_{\text{basal}}^2 + 2m_{\text{size}}\left(\sqrt{e^{\beta t_{j-1}}S_0} + 4\sqrt{e^{\beta t_j}S_0}\right)m_{\text{basal}}\right.\\
&\quad\left.\left.+2m_{\text{size}}^2\sqrt{e^{\beta t_{j-1}}S_0}\sqrt{e^{\beta t_j}S_0}\right)\right]\\
&- 10\beta^4\left(e^{d_{\text{metas}}t_{j-1}} - e^{d_{\text{metas}}t_j}\right)^4 m_{\text{basal}}^4 d_{\text{metas}}\left[e^{d_{\text{metas}}t_{j-1}}\left(m_{\text{basal}} + m_{\text{size}}\sqrt{e^{\beta t_{j-1}}S_0}\right)\right.\\
&\quad\left.-e^{d_{\text{metas}}t_j}\left(m_{\text{basal}} + m_{\text{size}}\sqrt{e^{\beta t_j}S_0}\right)\right]\\
&- \beta^5\left(e^{d_{\text{metas}}t_{j-1}} - e^{d_{\text{metas}}t_j}\right)^5 m_{\text{basal}}^5\Bigg)
\end{aligned}
$$

References

Engel J, Eckel R, Kerr J, Schmidt M, Fürstenberger G, Richter R, Sauer H, Senn HJ, Hölzel D (2003) The process of metastasisation for breast cancer. European journal of cancer 39(12):1794–1806

Gabbiani F, Cox SJ (2010) Stochastic Processes. In: Mathematics for Neuroscientists. Elsevier, p 251–266
